# Cellular and Extracellular Proteome of the Animal Pathogen *Corynebacterium silvaticum*, a Close Relative of Zoonotic *Corynebacterium ulcerans* and *Corynebacterium pseudotuberculosis*

**DOI:** 10.3390/proteomes8030019

**Published:** 2020-08-12

**Authors:** Jens Möller, Svenja Schorlemmer, Jörg Hofmann, Andreas Burkovski

**Affiliations:** 1 Microbiology Division, Department of Biology, Friedrich-Alexander-Universität Erlangen-Nürnberg, Staudtstr. 5, 91058 Erlangen, Germany; jens.moeller@fau.de (J.M.); svenja.schorlemmer@gmail.com (S.S.); 2 Biochemistry Division, Department of Biology, Friedrich-Alexander-Universität Erlangen-Nürnberg, Staudtstr. 5, 91058 Erlangen, Germany; joerg.hofmann@fau.de

**Keywords:** exoproteome, LC-MS/MS, mass spectrometry, necrotizing lymphadenitis, secretome, trypsin-like serine protease, zoonosis

## Abstract

*Corynebacterium silvaticum* is a newly described animal pathogen, closely related to the emerging human pathogen *Corynebacterium ulcerans* and *Corynebacterium pseudotuberculosis*, a major pathogen of small ruminants. In this study, proteins of a whole cell and a shaving fraction and the exoproteome of *C. silvaticum* strain W25 were analyzed as a first proteome study of this species. In total, 1305 proteins were identified out of 2013 proteins encoded by the W25 genome sequence and number of putative virulence factors were detected already under standard growth conditions including phospholipase D and sialidase. An up to now uncharacterized trypsin-like protease is by far the most secreted protein in this species, indicating a putative role in pathogenicity. Furthermore, the proteome analyses carried out in this study support the recently published taxonomical delineation of *C. silvaticum* from the closely related zoonotic *Corynebacterium* species.

## 1. Introduction

The genus *Corynebacterium*, first described in 1886 as a group of Gram-positive, club- (greek: *korune*) or rod-shaped bacteria [1], comprises between 142 and 145 different species today [2,3]. These were often isolated from human or animal material and are frequently of medical or veterinary importance [4,5]. *Corynebacterium diphtheriae*, the type strain of the genus, is the etiological agent of diphtheria [6,7,8] and most likely the best investigated pathogenic *Corynebacterium* species due to its life-threatening properties [9]. Already in 1884, Loeffler identified *C. diphtheriae* as etiological agent of diphtheria and postulated that a toxin secreted by the pathogen is responsible for the often fatal damage on different organs [10]. In fact, diphtheria toxin is the main virulence factor of *C. diphtheriae* and responsible for the life-threatening symptoms of respiratory diphtheria [11,12].

Interestingly, also the closely related *Corynebacterium ulcerans*, which was first described in 1927 [13], can evoke diphtheria-like symptoms. In fact, cases of diphtheria associated with *C. ulcerans* infections have outnumbered cases attributed to *C. diphtheriae* in European countries [14] and consequently *C. ulcerans* is recognized as an emerging pathogen today [15,16]. *C. ulcerans* infections of humans are almost exclusively the result of zoonotic transmission. Animal hosts range from farm animals such as cattle, goats and pigs to pets such as cats and dogs and wild animals, e.g., camels, hedgehogs, monkeys, orcas, otters, and water rats [16,17].

*C. diphtheriae* and *C. ulcerans* were clustered together with a third species, *Corynebacterium pseudotuberculosis*, in the group of toxigenic corynebacterial [18], which are characterized by the fact that they can be lysogenized by *tox* gene carrying corynephages and consequently express diphtheria toxin [19]. *C. pseudotuberculosis* is an important pathogen of small ruminants such as sheep and goat and causes severe economic losses [20]. Typically, *C. pseudotuberculosis* infections are characterized by abscesses and caseous lymphadenitis.

Recently the genome sequence of the atypical *C. ulcerans* strain W25, isolated from a wild boar with necrotizing lymphadenitis, was published [21]. Taxonomic analyses of the genome sequence data revealed that this isolate is clustered with two different isolates previously annotated as *C. ulcerans* KL1196 (identical to CVUAS6455 [17]) and *C. pseudotuberculosis* PO100/5 [22] and clearly delineated from other published *C. ulcerans* strains [23]. In parallel to this study, based on biochemical properties such as carbon metabolism, antibiotic resistance pattern and lipid profile in combination with 16SrRNA gene and *rpoB* sequence analysis, a new species, *Corynebacterium silvaticum*, was described recently [3] formed from former *C. ulcerans* strains isolated from forest-dwelling game animals, e.g., roe deer and wild boar. Strain W25, a non-toxigenic *tox* gene bearing (NTTB) strain [23], is obviously a member of this newly validated species.

Since we are interested in the analysis of pathogenicity determinants of corynebacteria [24,25,26,27] and different aspects of bacterial proteomics [28,29,30,31,32,33,34,35,36,37,38,39,40,41,42], we started a characterization of the cellular and extracellular proteome of *C. silvaticum* isolate W25, which is presented here. In total, 1305 proteins were identified out of 2013 proteins encoded by the W25 genome sequence including a number of putative virulence factors such as phospholipase D and sialidase.

## 2. Materials and Methods

### 2.1. Strains and Growth Conditions

*C. silvaticum* W25 [21,23] was grown in Brain Heart Infusion (BHI) supplemented with 0.05 M Tween 80 and 10% Fetal Calf Serum under constant shaking (125 rpm) at 37 °C. For preparation of proteins, bacteria were harvested by centrifugation (4000× *g*, 5 min), washed with PBS and used to inoculate 100 mL of BHI without supplements to an OD_600_ of 0.15. The bacteria were grown for 3 h until the exponential phase (OD_600_ approximately 0.6) was reached.

### 2.2. Sample Preparation for Mass Spectrometric Analyses

Extracellular proteins were prepared as described [31]. Cells were removed by centrifugation (10 min, 4 °C, 4000× *g*) and the supernatant was subsequently filtered using a 0.2 µm pore size filter (Minisart, Sartorius, Göttingen, Germany). After TCA precipitation the proteins were resuspended in protein buffer (10 mM DTT, 2% sodium deoxycholate, 50 mM Tris, pH 8.0). Protein concentration was determined using Pierce^TM^ 660 nm Protein Assay (Thermo Fisher Scientific, Bremen, Germany). Tryptic digest of 25 µg of the protein samples was carried out on 10 kDa vivacon 500 membrane filters as previously described following a modified Filter Aided Sample preparation (FASP) protocol [32,43,44].

For isolation of cell surface proteins by tryptic shaving [31,45] independent batches of cells were harvested and treated with 1.5 µg sequencing grade trypsin (Promega, Madison, WI, USA) for 1.5 h at 37 °C.

For analysis of the whole proteome the cells were resuspended in PBS buffer with protease inhibitor (Roche, Basel, Switzerland) and lysed with a homogenizer (MP Biomedical, Fisher Scientific, Schwerte, Germany) using glass beads (5.5 m s^−1^, 30 s, 5 cycles, 4 °C). To remove the cell debris the samples were centrifuged at 13,000× *g* for 30 min at 4 °C and the supernatant were retained. Protein buffer was added to a final concentration of 10 mM DTT, 2% sodium deoxycholate and 50 mM Tris (pH 8.0) and 40 µg of these proteins were transferred into a new low-binding tube. An incubation at 55 °C for 30 min was applied for protein denaturation followed by an alkylation of sulfhydryl groups (40 mM CAA final concentration). Proteins were precipitated overnight at 4 °C with 80% acetone, washed three times with 80% ice-cold acetone and dried under N_2_ atmosphere to avoid oxidation [44]. The resulting protein pellet was resuspended in 100 mM TEAB buffer with 2 µg of trypsin and digested overnight at 37 °C under constant shaking. A clean-up with C18 stage tips was performed to desalt 25 µg of all samples (three biological replicates of secreted proteins, whole proteome and surface proteins). Peptides were vacuum-dried and resuspended in 0.1% trifluoroacetic acid (TFA) for LC-MS/MS analysis [31].

### 2.3. Mass Spectrometric Analyses

Mass spectrometric analyses were carried out following a previously published protocol [31,44]. Ten µg of peptides were loaded onto a nanoflow Ultimate 3000 HPLC (Dionex, Sunnyvale, CA, USA). For separation of peptides an EASY-Spray column (Thermo Fisher Scientific; C18 with 2 µm particle size, 50 cm × 75 µm) was used (flow rate: 200 nL min^−1^; increasing acetonitrile concentrations over 120 min). Total method duration (including equilibration and column wash) was 160 min. Analyses of all samples were carried out using an Orbitrap Fusion mass spectrometer (Thermo Fisher Scientific, Bremen, Germany). Settings were adjusted according to a recently published protocol [32]: spray voltage 2000 V, transfer tube temperature 275 °C, scan range for the MS 1 detection in the Orbitrap 300–2000 (m/z), 50 ms maximum injection time, automatic gain control (AGC) target of 400,000 and Orbitrap resolution of 120.000. The most intense ions were selected for collision-induced dissociation with collision energy of 35%. For ion trap detection a maximum injection time of 250 ms and an AGC target of 100 were set [31,44]. Resulting raw data files were analyzed using the Proteome Discoverer 1.4 program package (Thermo Fisher Scientific, Bremen, Germany) and the W25 database (Proteome Id: UP000311124) in UniProt (www.uniprot/proteomes). The theoretical masses of peptides were generated with a maximum of two missed cleavages, as described in [46]. Carbamidomethyl modification on cysteine was set as fixed modification, oxidation of methionine as dynamic modification. Mass tolerance for survey scans was set to 10 ppm and 0.6 Da for fragment mass measurements, to compare the measured spectra of product ions. False discovery rate (FDR) was set on 1% for protein identification.

### 2.4. Bioinformatic Analyses

Information on localizations of proteins was extracted from Uniprot [47]. Proteins with unknown localization were analysed using the psortb server [48]. Quantification of proteins within the samples were carried using the total protein approach (TPA) method [49]. For unknown proteins, BLASTp was used to search for homologous proteins. For pathway analysis, UniProt IDs of identified proteins were used for a BlastKOALA search [50]. Proteome comparison was carried out using the PATRIC 3.6.5 database [51].

### 2.5. Reverse CAMP Test

For detection of PLD secretion a reverse CAMP test was carried out [31] using *Staphylococcus aureus* strain ATCC 29,213 as hemolytic pathogen and *C. ulcerans* strains 809 and BR-AD22, *C. silvaticum* W25 and *C. pseudotuberculosis* FRC41 to test PLD activity. Incubation was carried out at different temperatures (room temperature, 30 °C, 37 °C, and 40 °C) for two days. Plates were stored overnight at 4 °C for better visibility of the halo resulting from hemolysis by *S. aureus*.

### 2.6. Data Availability Statement

Mass spectrometry results have been deposited to the ProteomeXchange Consortium (http://proteomecentral.proteomexchange.org) via the PRIDE partner repository [52]. Data are available via ProteomeXchange with PXD020400.

## 3. Results

### 3.1. Analysis of Cellular Proteins

**Figure 1:**
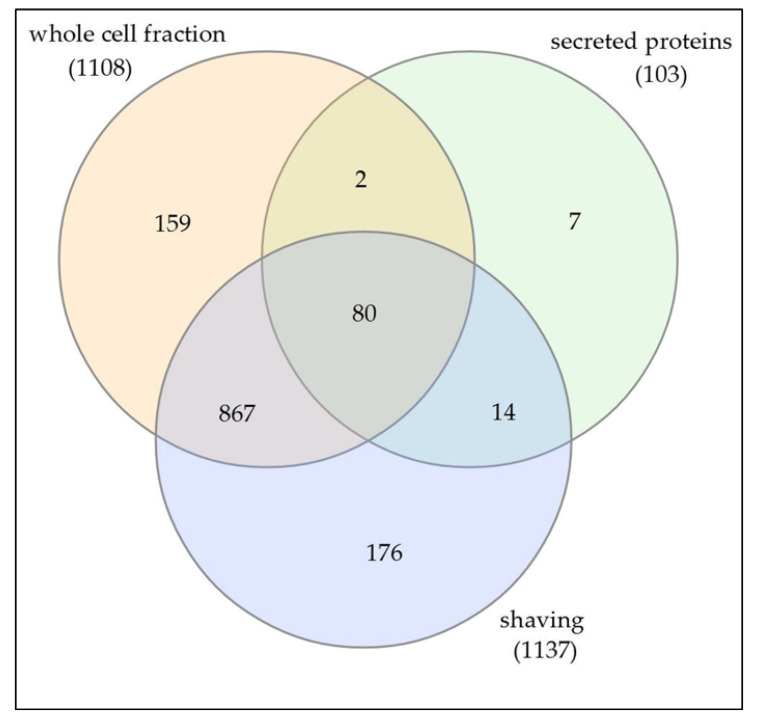
Distribution of identified proteins in whole cell, secreted and shaving fraction.

The theoretical proteome of *C. silvaticum* strain W25 comprises 2013 different proteins. In this study, we were able to detect 1305 proteins or 64.8% of the total theoretical proteome when cells were grown in complex medium. When protein fractions prepared in this study were analyzed, the whole cell fraction of 1108 proteins showed an overlap of 94 proteins (8.5%) with the secreted proteins. Seven proteins (0.5% of all proteins identified) were only found in the medium fraction. The trypsin shaving fraction comprised 1137 proteins with an overlap of 947 proteins with the whole cell fractions, indicating a massive cell lysis of strain W25 due to protease treatment. A similar effect was observed for *C. ulcerans* strain BR-AD22 before [31]. Nevertheless, 190 proteins (9.4% of all proteins identified) were additionally observed after trypsin shaving of the bacterial cells. The secreted protein fraction comprised 103 proteins (5.1% of all proteins) with an overlap of 94 proteins with the shaving fraction (91.3%) and 82 proteins (79.6%) with the whole cell fraction (Figure 1).

### 3.2. Characterization of Whole Cell and Shaving Fraction

**Figure 2:**
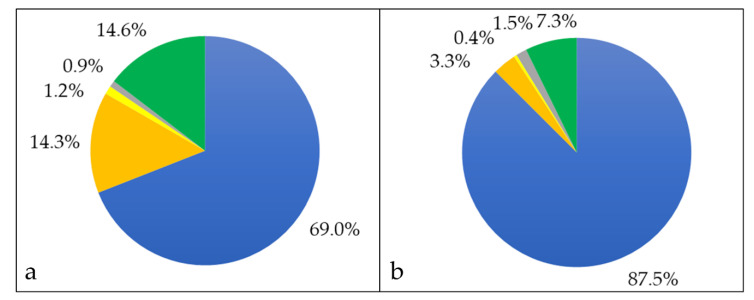
Whole cell proteome of *C. silvaticum* strain W25. Percentage of different proteins in cytoplasmic (blue), cytoplasmic membrane (orange), cell wall (yellow), extracellular (grey) and unknown (green) fraction. (**a**) The percentages in respect to the number of proteins and (**b**) in respect to their abundance are shown (see also Supplementary Material Table S1).

When the proteins of the whole cell fraction were analyzed in respect to their localization, more than two thirds (765 proteins, 69%) were cytoplasmic proteins, 158 proteins (14.3%) were attributed to the cytoplasmic membrane, 10 (0.9%) were cell wall-located and 13 (1.2%) were annotated as secreted, while for 162 proteins (14.6%) the putative localization was unknown (Figure 2a). When analyzed in respect to protein abundance, cytoplasmic proteins corresponded to 87.5% of the total proteins in the whole cell fraction, cytoplasmic membrane proteins to 3.3%, extracellular proteins to 1.5%, cell wall proteins to 0.4% and proteins with unknown localization to 7.3% (Figure 2b).

**Figure 3:**
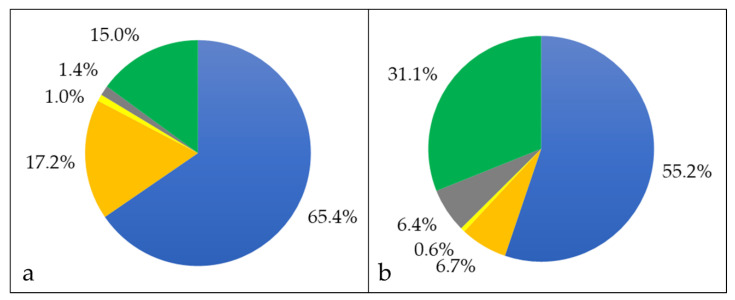
Proteins exclusively found in the shaving fraction. Percentage of different proteins in cytoplasmic (blue), cytoplasmic membrane (orange), cell wall (yellow), extracellular (grey) and unknown (green) fraction. (**a**) The percentages in respect to the number of proteins and (**b**) in respect to their abundance are shown.

Bacterial surface proteins may be shaved from the cells using trypsin [45]. In case of *C. silvaticum* W25, an unusual high number of 1137 proteins were observed, even exceeding the whole cell proteome (see also Supplementary Material Table S2). Obviously, protease treatment destabilizes the *C. silvaticum* cell wall structure leading to cell lysis as observed for a *C. ulcerans* strain before [31]. Nevertheless, in addition to the whole cell fraction, cell wall-anchored proteins were released by trypsin treatment and became accessible for mass spectrometry analysis. In the shaving fraction, 744 cytoplasmic proteins were annotated, corresponding to 65.4% of the total fraction, and 196 proteins were attributed to the cytoplasmic membrane including solute binding components of bacterial ABC transporters, which are known to be accessible from the outside in corynebacteria [53]. Sixteen proteins with extracellular localization, 11 located to the bacterial cell wall and 170 proteins without known localization (Figure 3a).

When the abundance of proteins exclusively found in the shaving fraction was analyzed in more detail, 5 proteins contributed to about 45.5% of the protein content, while the remaining 180 proteins sum up to 54.5% (see also Supplementary Material Table S3). With 15.5% the main protein observed was an uncharacterized protein, followed by an esterase family protein (10.0%), a DUF2505 domain-containing protein (9.4%) a cell wall channel (6.4%), and a DUF4185 domain-containing protein (4.2%).

### 3.3. Analysis of Extracellular Proteins

**Figure 4:**
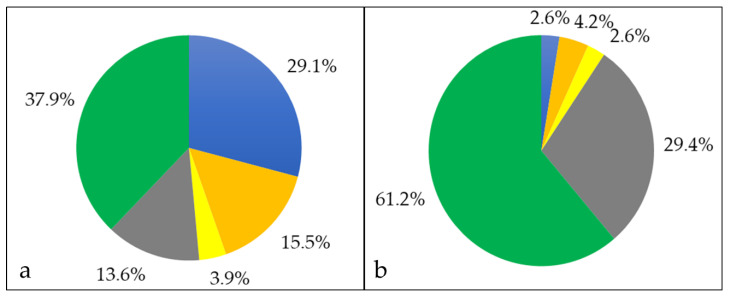
Extracellular proteome of *C. silvaticum* strain W25. Percentage of different proteins in cytoplasmic (blue), cytoplasmic membrane (orange), cell wall (yellow), extracellular (grey) and unknown (green) fraction. (**a**) The percentages in respect to the number of proteins and (**b**) in respect to their abundance are shown (see also Supplementary Material Table S4).

When the proteins secreted into the growth medium were harvested and analyzed, 103 proteins were identified. In this fraction, 30 proteins had a cytoplasmic localization, 16 proteins were membrane bound, 14 proteins were extracellular, 4 proteins are located to the cell wall, and 39 with unknown annotation (Figure 4).

Quantification of the proteins annotated as extracellular revealed that five proteins sum up to 95.7% of the total protein content, 88.1% were attributed to a single uncharacterized protein with the identifier A0A5C5F2T7, 2.2% to a hydrolase with the identifier A0A5C5F0I9 and A0A5C5F4U0, respectively, 1.9% to an M23 family metallopeptidase (A0A5C5F5R9) and 1.3% to another hydrolase (A0A5F0A739) (see also Supplementary Material Table S5).

Bioinformatic analyses of the major secreted protein showed that the uncharacterized and putative secreted protein A0A5C5F2T7 contains a signal peptide (aa 1–24), three disorder domains (aa 23–32, aa 164–189 and 347–357) two globular domains (aa 72–163 and aa 190–346), two low-complexity regions (aa2 59–275 and aa 352–363) and coiled-coil region (aa 282–349). A function prediction using the online tool Phyre2 showed homologous proteins annotated as proteases, trypsin-like serine proteases or hydrolases. A blastp search revealed a homologous protein in *C. diphtheriae* DIP2069 with 52.2% identity and identical predicted function.

### 3.4. Metabolic Pathway Analysis

**Figure 5:**
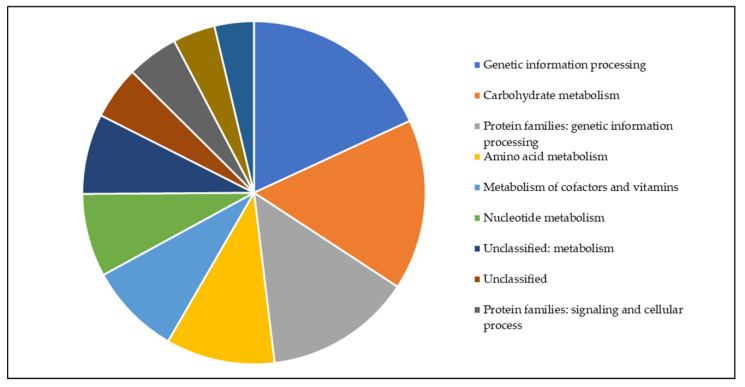
Functional categories of W25 proteins. All proteins found in this study were analyzed using BlastKoala and are shown as a pie chart (for color code, see inset).

For a first analysis of identified proteins from *C. silvaticum* W25 grown under full medium conditions a BlastKOALA search was carried out. Proteins of the main metabolic pathways (carbohydrate, fatty acid, nucleotide, amino acid, energy metabolism, and metabolism of cofactors and vitamins) were found (Figure 5).

Since *C. silvaticum* strain W25 was grown in Brain Heart Infusion, which contains not only glucose but also a high variety of different peptides and amino acids, we analyzed sugar and amino acid degradation pathways. Proteins for the complete glycolysis and pentose phosphate pathways were found (see Supplementary Material Table S6) and enzymes for the degradation of alanine, aspartate, asparagine glutamate glutamine, and serine were found (see Supplementary Material Table S7).

**Figure 6:**
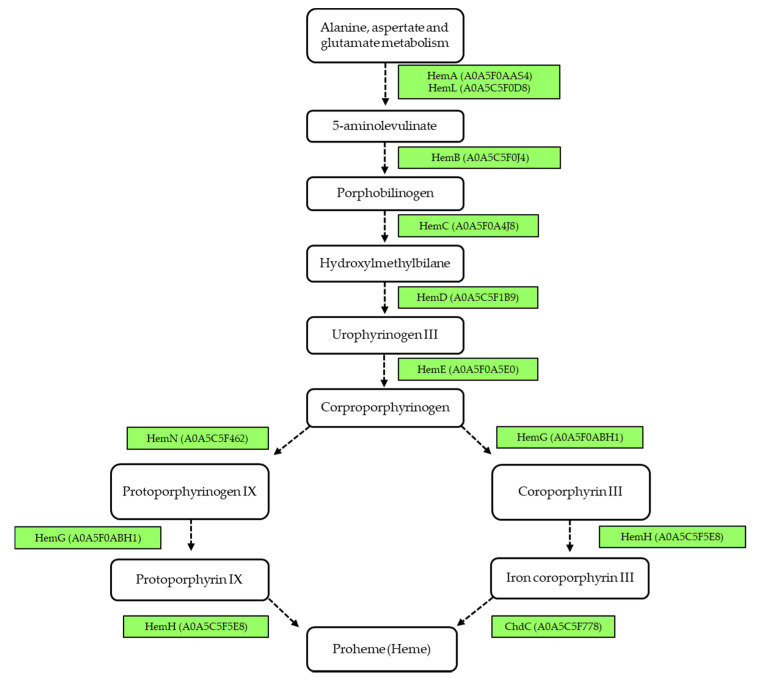
Heme biosynthesis in *Corynebacterium silvaticum* W25. Schematic overview shows intermediates, enzymes and the corresponding UniProt IDs (in brackets) according to the protoporphyrin-dependent (adapted from Kegg) and coproporphyrinogen-dependent pathway (adapted from [54]). Enzymes in green were identified in the whole cell fraction of W25.

In addition, we analyzed the biosynthesis pathway of heme as a co-factor, which is characteristic for corynebacteria. Besides the common protoporphyrin-dependent pathway, an alternative coproporphyrinogen-dependent pathway was predicted recently [54]. The key protein of this pathway, coproheme decarboxylase (ChdC), was identified due to its similarity to DIP1394 among the observed proteins in this study, annotated as chlorite dismutase family protein A0A5C5F778. Heme biosynthesis pathways in *C. silvaticum* are shown in Figure 6.

### 3.5. Analysis of Virulence Factors

From the genome sequence of *C. silvaticum* W25, a number of putative virulence factors was annotated [23]. In this study 12 of these were detected, while 3 were not found (Table 1).

**Figure 7:**
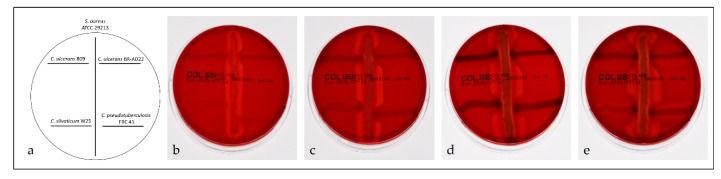
Reverse CAMP test. Hemolysis by *S. aureus* strain ATCC 29,213 is inhibited by *C. ulcerans* strains BR-AD22, 809, *C. silvaticum* W25 and *C. pseudotuberculosis* strain FRC41 as indicated by arrowhead-like indentations of the hemolysis halos. (**a**) application scheme; incubation at (**b**) room temperature, (**c**) 30 °C, (**d**) 37 °C, and (**e**) 40 °C.

Phospholipase D (PLD) is the major virulence factor of *C. pseudotuberculosis* [20,55,56] and also found in *C. ulcerans* [16]. Since phospholipase D was observed exclusively in the shaving fraction in this study, we analyzed the activity of the enzyme in a reverse CAMP test (Figure 7). Interestingly, a temperature-dependent secretion of the enzyme was observed, which was negligible at room temperature and highest at 40 °C. Activity of the enzyme was estimated by the effect on hemolysis activity of *S. aureus* (halo formation) and not strictly dependent on growth.

## 4. Discussion

The study presented here provides the first and basic characterization of the cellular and extracellular proteome of the animal pathogen *C. silvaticum*. Besides proteins with annotated function, a number of unknown and functionally not annotated or hypothetical proteins were found, which emphasizes that proteome analyses may be helpful to characterize the expression and localization of proteins even when only a very basic genome annotation is available. This was especially exemplified by the characterization of A0A5C5F2T7, annotated as putative secreted protein. In this study, we showed that A0A5C5F2T7 is not only expressed, but is by far the major secreted protein of *C. silvaticum*. Although further experimental data are missing, bioinformatic analyses hint to a protease function. Two other virulence factors Vsp1 and Vsp2 contain a similar trypsin-like serine protease domain as A0A5C5F2T7, which may indicate a prominent role in pathogenicity. Another protein (A0A5F0A739) of the five most abundant proteins of the extracellular proteome may also be associated with pathogenicity. The annotated hydrolase A0A5F0A739 contains a NlpC/P60 domain. Proteins of this superfamily are highly conserved among corynebacteria [26]. A study by Hansmeier and co-workers identified an invasion-associated protein DIP1281 a NlpC/P60 protein member in the secretome of *C. diphtheriae* [57]. It is assumed that proteins of this family play a fundamental role in corynebacterial cell surface organization, cell separation, adhesion, and internalization in host cells [26,58]. *C. silvaticum* strain W25 produced almost all identified virulence factors [23] already under standard growth conditions except Vsp1, Cpp and NrpS1. The presence of two proteins in the extracellular environment with putative virulence potential (A0A5C5F2T7 and A0A5F0A739) needs to be further investigated.

*C. silvaticum* is considered as a pathogen of forest-dwelling animal. In respect to host-pathogen interaction, not only the number of putative virulence factors detected in this study, but also the high abundant surface and secreted proteins may be of interest for further studies. Growth tests at different temperatures and analysis of PLD activity indicate that *C. silvaticum* growth and virulence is strictly dependent on temperatures around 37 °C, which fits with its isolation from abscesses in wild boar and roe deer. Furthermore, from this temperature profile, it may be expected that *C. silvaticum* will not cause skin infections as found for *C. ulcerans* and *C. diphtheriae* [15]. Furthermore, *C. ulcerans* 809 seem to have a temperature-regulated PLD expression, which solves the question as to why no PLD protein and activity was found in a previous study [31].

**Figure 8:**
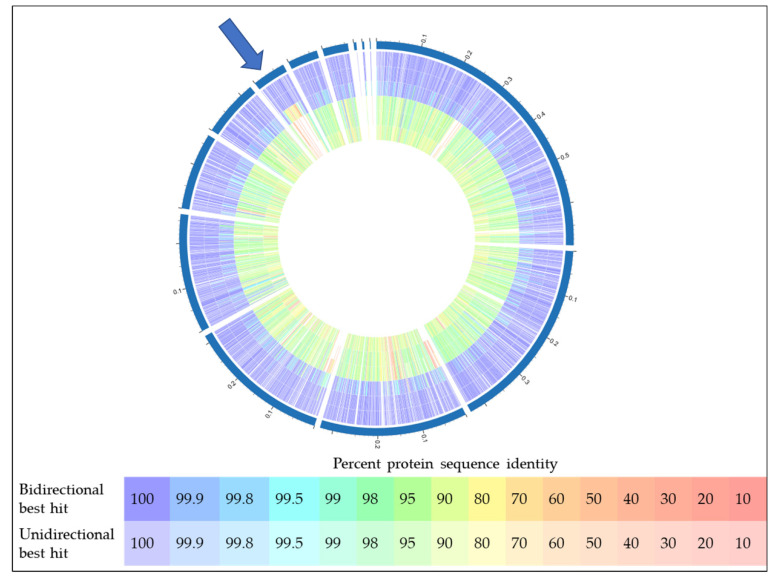
Proteome comparison of different *Corynebacterium* species. From outside to inside, 1: *C. silvaticum* W25, 2: *C. silvaticum* KL1196, 3: *C. silvaticum* PO100/5, 4: *C. ulcerans* BR-AD22, 5: *C. ulcerans* 809, 6: *C. pseudotuberculosis* FRC41. The blue arrow indicates a prophage region. For color code, see inset.

In addition to the experimental work described above, bioinformatics analyses of the predicted W25 proteome were carried out, which support the recently published taxonomical delineation of *C. silvaticum* from the closely related zoonotic *Corynebacterium* species *C. ulcerans* and *C. pseudotuberculosis*. When the proteome of strain W25 was compared with those of *C. silvaticum* strains KL1196 and PO100/5, a high identity throughout the proteins was found. Interestingly, the PO100/5 isolate originating from Portugal seems to be less conserved compared to the *C. silvaticum* isolates from Germany, W25 and KL1196. Within this analysis, *C. ulcerans* strains BR-AD22 and 809 [59] were easily distinguishable from this cluster and are more related to *C. pseudotuberculosis* FRC41 [60] (Figure 8). One major difference between PO100/5 and strains W25 and KL1196 is the presence of a prophage region, also detectable in the *C. ulcerans* and *C. pseudotuberculosis* strains used in this study (indicated in red and by an arrow in Figure 8).

In addition, proteome comparisons revealed 320 proteins exclusively found in *C. silvaticum* species, 208 proteins being annotated as hypothetical proteins and 19 proteins being phage-associated. Within the remaining 93 proteins, 10 proteins are involved in peptide binding and protein transport (see Supplementary Material, Table S8), an observation, which may indicate a high importance of amino acids and peptides as nutrient source for *C. silvaticum*.

## 5. Conclusions

In this study, a first proteome analysis of *C. silvaticum* strain W25 was carried out and almost two-thirds of the proteins encoded in the genome sequence were detected including a number of virulence factors. Analysis of the extracellular proteome indicated a putative role of an up to now uncharacterized trypsin-like protease in pathogenicity. Furthermore, the proteome analyses carried out support the recently published taxonomical delineation of *C. silvaticum* from the closely related zoonotic *Corynebacterium* species.

## Figures and Tables

**Table 1:** Virulence factors of *C. silvaticum* W25. Protein and gene designations are indicated together with the fraction in which the proteins were found (+: present, −: absent). Absence of the corresponding protein in all fractions analyzed is indicated in bold.

Protein	Gene	Whole Cell	Secreted	Shaving
Phospholipase D	*pld*	**−**	−	**+**
Neuraminidase (sialidase)	*nanH*	**+**	**+**	**+**
**Trypsin-like serine protease**	***vsp1***	**−**	**−**	**−**
Peptidoglycan endopeptidase	*ripA*	**+**	**+**	**+**
Hydrolase (cell wall peptidase)	*cwlH*	**+**	**+**	**+**
Protease	*vsp2*	**+**	**+**	−
**Protease (endo-beta-N-acetylglucosaminidase F2)**	***cpp***	**−**	**−**	**−**
Type VII secretion-associated serine protease mycosin	*cpfrc_00397*	**+**	−	−
AccD5-3 acyl-CoA carboxylase beta	*dtsR2*	**+**	−	**+**
AccD5-2 propionyl-CoA carboxylase beta chain 2	*dtsR1*	**+**	−	**+**
AccD5-1 acyl-CoA carboxylase subunit beta	*accD3*	**+**	−	**+**
Hydrolase	*cpfrc_00536*	**+**	−	**+**
**Non-ribosomal peptide synthetase**	***nrpS1***	**−**	**−**	**−**
Resuscitation-promoting factor	*rpfA*	+	+	+
Resuscitation-promoting factor	*rpfB*	+	+	+

## Supplementary Materials

The following are available online at https://www.mdpi.com/2227-7382/8/3/19/s1, Table S1. Identified proteins in the whole cell fraction. The table shows the Uniprot ID, description, molecular weight number of amino acids, the area and quantification of three biological replicates. Table S2. Identified proteins of the shaving fraction. The table shows the Uniprot ID, description, molecular weight number of amino acids, the area and quantification of three biological replicates. Table S3. Selected proteins of the shaving fraction with predicted localization ot cell surface. The table shows the Uniprot ID, description, molecular weight number of amino acids, the area and quantification of three biological replicates. Table S4. Identified proteins of the extracellular proteome. The table shows the Uniprot ID, description, molecular weight number of amino acids, the area and quantification of three biological replicates. Table S5. Selected proteins of the extracellular proteome with predicted extracellular localization. The table shows the Uniprot ID, description, molecular weight number of amino acids, the area and quantification of three biological replicates. Table S6. Glycolysis and pentose phosphate pathway. The table gives the Kegg ID, corresponding UniProt ID and the protein name. Table S7. Enzymes involved in alanine, aspertate, asparagine, glutamate, glutamine and serine metabolism. Table S8. Proteins execlusively found in C. silvaticum spezies. Phage related proteins are highlited in red, hypothetical proteins in yellow.
